# A Multicenter Validation of a Fully Automated Chemiluminescence Immunoassay for Plasma Elafin in Psoriasis Diagnosis and Severity Assessment

**DOI:** 10.1002/advs.202511535

**Published:** 2025-09-30

**Authors:** Jingkun Yi, Meng Xu, Ling Han, Zhining Dong, Ruimin Liu, Xiaomei Zhang, Di Hu, Chuanjian Lu, Xiaobo Yu

**Affiliations:** ^1^ State Key Laboratory of Medical Proteomics Beijing Proteome Research Center National Center for Protein Sciences‐Beijing (PHOENIX Center) Beijing Institute of Lifeomics Beijing 102206 China; ^2^ Department of Biomedical Informatics State Key Laboratory of Vascular Homeostasis and Remodeling School of Basic Medical Sciences Peking University Beijing 100191 China; ^3^ Department of Clinical Laboratory, The First Affiliated Hospital of Zhengzhou University Zhengzhou Henan 450052 China; ^4^ The Second Clinical College of Guangzhou University of Chinese Medicine Guangzhou Guangdong 510000 China; ^5^ Chinese Medicine Guangdong Laboratory Hengqin Guangdong 519000 China; ^6^ State Key Laboratory of Dampness Syndrome of Chinese Medicine The Second Affiliated Hospital of Guangzhou University of Chinese Medicine Guangzhou Guangdong 510120 China; ^7^ Guangdong Provincial Key Laboratory of Clinical Research on Traditional Chinese Medicine Syndrome Guangzhou Guangdong 510000 China; ^8^ Guangdong‐Hong Kong‐Macau Joint Lab on Chinese Medicine and Immune Disease Research Guangzhou University of Chinese Medicine Guangzhou Guangdong 510120 China; ^9^ Guangzhou DARUl Biotechnology Co. Ltd Guangzhou Guangdong 510000 China; ^10^ Hebei University Baoding Hebei 071002 China; ^11^ ProteomicsEra Medical Co., Ltd Beijing 102206 China; ^12^ State Key Laboratory of Traditional Chinese Medicine Syndrome the Second Affiliated Hospital of Guangzhou University of Chinese Medicine Guangzhou Guangdong 510120 China; ^13^ School of Basic Medicine Sciences, Anhui Medical University, Hefei, Anhui, PR China. Hefei Anhui 230032 China; ^14^ Basic Medical College Qingdao University Qingdao 266071 China; ^15^ International Academy of Phronesis Medicine (Guangdong) Guangzhou 510320 China

**Keywords:** full‑course management, fully automated chemiluminescence immunoassays, psoriasis

## Abstract

Psoriasis diagnosis and severity assessment rely on subjective skin evaluations. Elafin is a promising biomarker, yet no high‐throughput clinical assay exists. A chemiluminescence immunoassay (CLIA) reagent kit for plasma Elafin has been developed and validated using automated analyzers to enable rapid diagnosis, objective severity stratification, and longitudinal monitoring. Analytical performance is rigorously assessed, including the limit of blank (LoB) (0.069 ng mL^−1^), limit of detection (LoD) (0.099 ng mL^−1^), limit of quantification (LoQ) (0.8 ng mL^−1^), linearity (0.8–200 ng mL^−1^), precision, recovery, reproducibility, and interference resistance. Plasma Elafin is quantified in 731 participants from five hospitals: 112 healthy controls, 466 psoriasis patients (mild = 164, moderate = 158, severe = 144), and 153 disease controls. The assay achieves 89% diagnostic accuracy with an area under the receiver operating characteristic curve (AUC) of 0.94 for psoriasis. It effectively distinguishes disease severity (AUC 0.68 mild vs moderate; 0.77 moderate vs severe), and Elafin levels correlated with treatment duration in moderate (R = 0.51) and severe (R = 0.41) cases. This CLIA reagent kit demonstrates excellent analytical and clinical performance, supporting automated, high‐throughput Elafin detection as a reliable tool for diagnosing and managing moderate‐to‐severe psoriasis.

## Graphical abstract

The fully automated CLIA demonstrates high sensitivity and specificity for plasma Elafin, achieving 88% diagnostic accuracy and an AUC of 0.92 for psoriasis detection in a multicenter cohort. The assay reliably distinguishes psoriasis from other dermatoses, objectively stratifies disease severity, and allows longitudinal monitoring, supporting its utility in clinical and research settings.

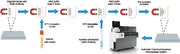

## 1 Introduction

Psoriasis is a common inflammatory dermatosis^[^ ^1^ ^]^ affecting ≈2–3% of the global population,^[^ ^2^ ^]^ with China experiencing a notably high disease burden.^[^ ^3^ ^]^ Its pathogenesis is multifactorial and remains incompletely understood, resulting in considerable variability in treatment responses among patients.^[^ ^4^ ^]^ Currently, psoriasis diagnosis primarily relies on clinical evaluation of skin manifestations such as scaling, pruritus, and erythema.^[^ ^5^, ^6^ ^]^ However, these assessments are subjective and may lead to misdiagnosis, especially when differentiating from similar dermatoses like atopic dermatitis and urticaria.^[^ ^7^ ^]^ Histopathological examination, although the diagnostic gold standard, is invasive, technically complex, and carries patient risks.^[^ ^7^ ^]^ Disease severity is typically quantified using the Psoriasis Area and Severity Index (PASI), assessing erythema, skin thickness, and scaling (each scored 0–4 points) weighted by affected body surface area.^[^ ^8^ ^]^ However, PASI scores are influenced by clinician subjectivity, complicated assessment procedures, and environmental factors such as temperature and humidity.^[^ ^9^ ^]^ Therefore, there is an urgent need for validated, non‐invasive biomarkers to facilitate rapid, accurate, and convenient clinical diagnosis and severity assessment.

Our previous proteomic studies identified Elafin as a promising biomarker for psoriasis, demonstrating significantly elevated plasma levels in patients compared to healthy controls, findings confirmed by Enzyme‐Linked Immunosorbent Assay (ELISA).^[^ ^10^ ^]^ Elafin, a serine elastase inhibitor derived from pre‐Elafin through proteolytic cleavage,^[^ ^11^ ^]^ was initially detected in psoriatic lesions in 1990.^[^ ^12^ ^]^ Its expression is significantly increased in psoriasis and correlates positively with disease severity, indicating potential diagnostic and prognostic value.^[^ ^9^, ^11^, ^13^, ^14^, ^15^, ^16^, ^17^, ^18^, ^19^ ^]^ Mechanistically, microbial dysbiosis in psoriasis induces keratinocytes to produce antimicrobial peptides (AMPs)—including Elafin—that recruit neutrophils and amplify cutaneous inflammation. Elafin functions dually as a neutrophil elastase–specific inhibitor and an AMP: on one hand, it restricts neutrophil elastase‐mediated tissue proteolysis and elastase‐induced neutrophil chemotaxis, thereby limiting neutrophil‐driven tissue damage and inflammation; on the other, its lesion‐specific overexpression in keratinocytes, particularly in the spinous layer, reflects a sustained inflammatory response.^[^ ^20^ ^]^ Transcriptional regulators such as CRABP2 modulate Elafin expression under dysbiosis, maintaining a pathological loop: microbial imbalance → AMP (Elafin) upregulation → neutrophil recruitment → pro‐inflammatory mediator release → keratinocyte hyperproliferation, driving chronic inflammation and hyperkeratosis.^[^ ^21^, ^22^ ^]^ This dual role highlights Elafin as both a compensatory anti‐protease and a marker of persistent inflammatory stress in psoriasis. Notably, Elafin is scarce in healthy skin but increases with disease severity, supporting its use as both a diagnostic marker and a dynamic indicator for disease monitoring.^[^ ^23^ ^]^

However, current ELISA‐based assays for Elafin are research‐oriented, exhibit low throughput, lengthy turnaround times, and lack international standardization, limiting their practical use in large‐scale clinical screening.^[^ ^24^ ^]^ In contrast, chemiluminescence immunoassays (CLIA) performed on automated platforms offer higher sensitivity, broader detection ranges, faster results, and better scalability, making them ideal for high‐throughput clinical diagnostics.^[^ ^25^, ^26^, ^27^, ^28^ ^]^ Therefore, CLIA reagent kits implemented on automated analyzers represent an optimal platform for plasma Elafin quantification, enabling high‐throughput screening and longitudinal monitoring of psoriasis.

This study aims to develop and validate a novel CLIA reagent kit for plasma Elafin detection using automated chemiluminescence analyzers—the first reagent kit designed specifically for rapid clinical diagnosis, severity assessment, and longitudinal psoriasis monitoring, thus promoting precision medicine. Rigorous quality control evaluations were conducted to ensure clinical applicability. The assay was validated using plasma samples from patients and healthy controls recruited from five hospitals: Hospital for Skin Diseases, Chinese Academy of Medical Sciences; Dermatology Hospital of Jiangxi Province; Liuzhou Worker's Hospital; Guangdong Provincial Hospital of Chinese Medicine and Guangzhou Red Cross Hospital. Our results demonstrate that this CLIA reagent kit is robust and clinically relevant, advancing precision medicine in psoriasis management.

## 2 Results

### 2.1 Development of the Fully Automated CLIA for Plasma Elafin Detection

The precision of the CLIA reagent kit was evaluated with two QC levels (C1: 25 ng mL^−1^; C2: 100 ng mL^−1^) on both DR‐CL2000 and Shine i1910 automated analyzers. Intra‐assay precision was assessed with ten replicates in a single run; inter‐assay precision was determined across five independent runs over nine days. Coefficients of variation (CVs) were <10% under all conditions, indicating acceptable repeatability and low total imprecision (**Table** **1**). Accuracy was verified by spike‐and‐recovery at 150 and 5 ng mL^−1^, yielding recoveries of 104.62% (DR‐CL2000) and 104.49% (Shine i1910), both within the acceptable 80%–120% range.

**Table 1:** Comparison of variations in plasma Elafin assay results across different instruments.

		Intra‐assay [N = 10]			Inter‐assay [N = 5]		
Samples	Instrument	Mean (ng/mL)	SD^a)^	CV (%)^a)^	Mean (ng/mL)	SD	CV (%)
C1	DR‐CL2000	25.30	0.56	2.21	22.16	1.15	5.19
C1	Shine i1910	25.19	0.73	2.90	23.35	0.53	2.27
C2	DR‐CL2000	101.55	3.86	3.80	95.52	3.10	3.25
C2	Shine i1910	100.91	3.14	3.11	92.25	7.08	7.67

^a)^ SD represents standard deviation and CV means coefficient of variation, which was calculated by the formula: CV = (SD/Mean) × 100%

**Figure 1:**
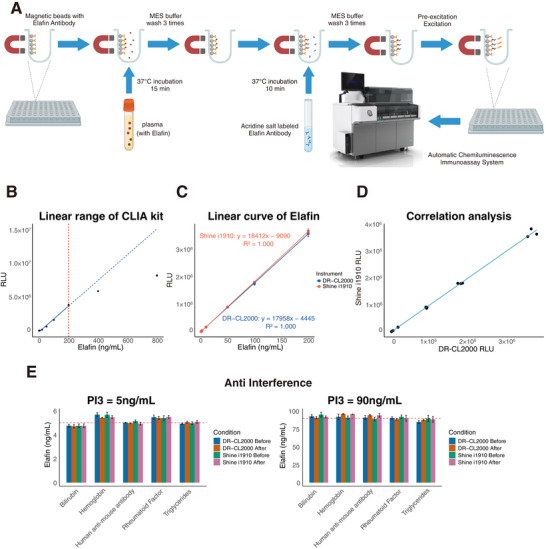
Development and quality control of the CLIA for plasma Elafin detection. A) Schematic workflow illustrating the construction of the CLIA kit. Created in BioRender. Yi, J. (2025) https://BioRender.com/iyxfid7 B) Linear range of the CLIA kit: The relative light units (RLU) showed a strong linear correlation with plasma Elafin concentrations below 200 ng mL^−1^. C) RLU measurements obtained from two automated analyzers (DR‐CL2000 and Shine i1910) within the linear range of the kit. D) High correlation of CLIA measurements between DR‐CL2000 and Shine i1910 analyzers. E) High concentrations of interfering substances in plasma do not affect the performance of the CLIA kit.

Analytical sensitivity was established by determining the limit of blank (LoB), limit of detection (LoD), and limit of quantification (LoQ) using appropriate statistical procedures. Linearity was evaluated from 0.8 to 800 ng mL^−1^; the analytical measurement range (AMR) was linear up to 200 ng mL^−1^, defining the upper limit of quantification (ULOQ) at 200 ng mL^−1^ (**Figure** **1**B) (**Table** **2**). High‐dose hook effect testing was performed at concentrations up to 2000 ng mL^−1^ to confirm assay reliability across the entire dynamic range. Subsequently, Elafin concentrations from 0.8 to 200 ng mL^−1^ were quantitatively measured using two chemiluminescence instruments, the DR‐CL2000 and Shine i1910 (Figure 1C,D). The experimental results showed that the linear correlation coefficient between the Elafin concentration obtained using the kit and the actual concentration exceeded 0.99, indicating that the kit has a good linear response for Elafin concentrations from 0.8 to 200 ng mL^−1^. Furthermore, to verify whether the CLIA kit exhibits a hook effect when measuring high concentration samples, Elafin samples at 2000 ng mL^−1^ were measured. The results from both instruments did not show a hook effect, and even at extremely high concentrations, the kit was able to provide accurate measurement results (Figure S1A,B, Supporting Information). This finding confirms that the kit maintains good stability and reliability across a wide concentration range, making it suitable for the accurate measurement of Elafin in clinical and research settings.

**Table 2:** The assay performance of the plasma Elafin CLIA assay.

Instrument	Recovery rate	LoB ^a)^	LoD ^a)^	LoQ ^a)^	ULOQ ^a)^	Dynamic Range
DR‐CL2000	104.62%	0.069 ng mL^−1^	0.096 ng mL^−1^	0.8 ng mL^−1^	200 ng mL^−1^	0.8–2000 ng mL^−1^
Shine i1910	104.49%	0.069 ng mL^−1^	0.099 ng mL^−1^	0.8 ng mL^−1^	200 ng mL^−1^	0.8–2000 ng mL^−1^

^a)^ LoB: limit of blank; LoD: limit of detection; LoQ: limit of quantification; ULOQ: upper limit of quantification

The kit is designed for detecting Elafin concentrations in plasma, so we paid particular attention to potential interfering factors that may exist in the plasma. To avoid the impact of abnormal blood biochemical markers such as hyperlipidemia and hemolysis, we selected the following substances as interferents: 1500 mg dL^−1^ triglycerides (TG) (normal plasma concentration in healthy individuals: <150 mg dL^−1^), 25 mg dL^−1^ bilirubin (STB) (normal plasma concentration in healthy individuals: 0.1–1.0 mg dL^−1^), 0.5 g dL^−1^ hemoglobin (HB) (normal plasma free hemoglobin concentration in healthy individuals: <5 mg/dL), 600 IU mL^−1^ rheumatoid factor (RF) (normal plasma concentration in healthy individuals: <20 IU/mL), and 500 ng mL^−1^ human anti‐mouse antibody (HAMA).^[^ ^29^, ^30^ ^]^ The results showed that there was no significant difference between the measured concentrations and actual concentrations before and after the addition of interferents, indicating that our kit can still accurately detect the corresponding Elafin concentration in the presence of high levels of interferent substances (Figure 1E). Therefore, this kit can reliably measure Elafin in blood samples containing interferents, providing a powerful tool for clinical diagnosis.

### 2.2 The Assay Performance of Plasma Elafin CLIA

**Figure 2:**
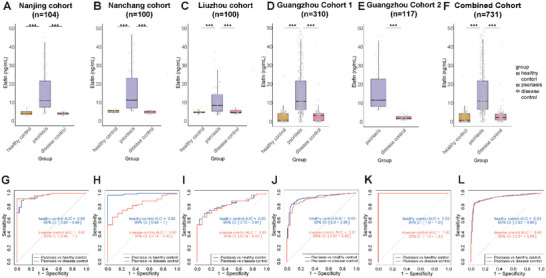
Diagnostic performance of the CLIA for plasma Elafin detection. A–F) Plasma Elafin concentrations in psoriatic patients were significantly higher compared to HC and DC in five individual cohorts (Nanjing, Nanchang, Liuzhou, Guangzhou Cohort 1, Guangzhou Cohort 2) as well as in the combined cohort. G–L) ROC curves indicate that the CLIA kit effectively discriminates psoriatic patients from healthy and disease control subjects in the five cohorts and the combined analysis. NS, not significant (*p* > 0.05); *p < 0.05, **p < 0.01, and ***p < 0.001 compared with Wilcoxon test.

We quantified plasma Elafin in 112 healthy controls (HC), 153 disease controls (DC), and 466 psoriasis patients (Pso) across five centers (Hospital for Skin Diseases, Chinese Academy of Medical Sciences; Dermatology Hospital of Jiangxi Province; Liuzhou Worker's Hospital; Guangdong Provincial Hospital of Chinese Medicine; Guangzhou Red Cross Hospital) to assess assay performance in psoriasis diagnosis. Most patient samples fell within the assay's linear dynamic range (0.8–200 ng mL^−1^) (Figure S2, Supporting Information). The optimal threshold for the Elafin kit was determined to be 6.01 ng mL^−1^ using receiver operating characteristic (ROC) curve analysis and Youden index calculation on all patients and healthy subjects from the five centers. Accordingly, a cutoff of 6.01 ng mL^−1^ was applied: values >6.01 ng mL^−1^ were classified as positive, ≤6.01 ng mL^−1^ as negative. Analyses were then performed per center. Among healthy subjects, stratification by age and sex showed no significant effects on Elafin (Table S1, Supporting Information). At the Hospital for Skin Diseases, Chinese Academy of Medical Sciences; Dermatology Hospital of Jiangxi Province; Liuzhou Worker's Hospital; Guangdong Provincial Hospital of Chinese Medicine; Guangzhou Red Cross Hospital, the overall accuracy between Elafin test results and classical diagnostic methods were 0.89, 0.98, 0.76, 0.88 and 1.00, respectively (**Table** **3**). The Elafin monitoring data for Pso at these five centers were significantly higher than those of healthy subjects (*p* < 0.001) (**Figure** **2**A–E), and area under the receiver operating characteristic curve (AUC) was 0.96, 0.99, 0.83, 0.93 and 1.00, respectively (Figure 2G–K). Between‐center heterogeneity was low for accuracy (I^2^ = 9%, *p* = 0.356), sensitivity (I^2^ = 4%, *p* = 0.385), specificity (I^2^ = 0%, *p* = 0.955), and AUC (I^2^ = 0%, *p* = 0.759), supporting fixed‐effect pooling (Table S2, Supporting Information). Pooled analysis across centers yielded an overall accuracy of 89%, sensitivity 86%, specificity 95% (Tables 3,**4**), and Kappa 0.774 (*p* < 0.001), reflecting moderate agreement with conventional diagnostics. The Elafin monitoring data for Pso from all five centers were significantly higher than HC (*p* < 0.001), with an AUC of 0.94 (Figure 2F,L).

**Table 3:** Multicenter performance of plasma Elafin CLIA for psoriasis detection.

	AUC [95% CI]^a)^	Accuracy [95% CI]	Sensitivity [95% CI]	Specificity [95% CI]
Hospital for Skin Diseases, Chinese Academy of Medical Sciences	0.96 (0.93–0.99)	0.89 (0.82–0.95)	0.89 (0.82–0.95)	0.89 (0.82–0.95)
Dermatology Hospital of Jiangxi Province	0.99 (0.98–1.00)	0.98 (0.93–1.00)	0.97 (0.90–1.00)	1.00 (0.88–1.00)
Liuzhou Worker's Hospital	0.83 (0.76–0.91)	0.76 (0.66–0.84)	0.73 (0.62–0.83)	0.82 (0.63–0.94)
Guangdong Provincial Hospital of Chinese Medicine	0.93 (0.90–0.96)	0.88 (0.84–0.91)	0.83 (0.65–0.89)	0.94 (0.84–0.98)
Guangzhou Red Cross Hospital	1.00 (1.00–1.00)	1.00 (0.97–1.00)	1.00 (0.95–1.00)	1.00 (0.93–1.00)
Total	0.94 (0.92–0.95)	0.89 (0.87–0.91)	0.86 (0.82–0.87)	0.95 (0.92–0.97)

^a)^ AUC: area under the receiver operating characteristic curve; CI: confidence interval

**Table 4:** Diagnostic accuracy of plasma Elafin CLIA for psoriasis detection.

		PASI		Total
		Positive^a)^	Negative	
Elafin	Positive	396	14	410
	Negative	66	255	321
Total		462	269	731

^a)^ Positive: PASI > 0

By analyzing the patients with discrepancies between the classical diagnostic method and the kit, it was found that most of the discrepant patients had received medication treatment within the past year, and the Elafin levels in the treatment group were lower than those in the untreated group (*p* < 0.001) (**Table** **5**). This indicates that recent pharmacotherapy alters Elafin levels. Excluding treated patients, assay accuracy improved to sensitivity 89% (95%CI 0.85–0.92), specificity 96% (95%CI 0.92–0.97), and accuracy 92% (95%CI 0.89–0.94). The Kappa value was 0.836 (*p* < 0.001), indicating good consistency between the kit and the classical diagnostic method (**Table** **6**).

**Table 5:** Plasma Elafin expression and PASI in Psoriasis with and without treatment.

	Group	Median (P25‐P75)^a)^
Elafin	treated	11.75 (6.74–32.11)
	untreated	18.63 (8.68–51.17)
PASI	treated	2.85 (1.80–8.97)
	untreated	6.60 (2.80–11.80)

^a)^ P25: 25th percentile (first quartile); P75: 75th percentile (third quartile)

**Table 6:** Diagnostic accuracy of plasma Elafin CLIA in medication‐naive psoriasis.

		PASI		Total
		Positive^a)^	Negative	
Elafin	Positive	282	12	294
	Negative	36	255	291
Total		318	267	585

^a)^ Positive: PASI > 0

To further evaluate whether treatment status affects the overall diagnostic performance of the assay, we conducted stratified analyses. Diagnostic accuracy was comparable between the total cohort (AUC = 0.94, sensitivity = 86%, specificity = 95%) and the treatment subgroup (AUC = 0.93, sensitivity = 79%, specificity = 95%), indicating that while treatment reduces absolute Elafin levels, it does not significantly compromise the diagnostic utility of the assay. These findings suggest that the CLIA reagent kit maintains reliable diagnostic performance across different treatment statuses, supporting its clinical applicability in real‐world settings where patients may have varied treatment histories.

Beyond Pso vs HC, the assay effectively differentiated Pso from DC. The Elafin test results for Pso at the Hospital for Skin Diseases, Chinese Academy of Medical Sciences; Dermatology Hospital of Jiangxi Province, Liuzhou Worker's Hospital, Guangdong Provincial Hospital of Chinese Medicine, and Guangzhou Red Cross Hospital were significantly higher than DC (*p* < 0.001) (Figure 2A–E). AUC for distinguishing between Pso and DC was 0.97, 0.99, 0.83, 0.91, and 1.00, respectively (Figure 2G–K). A joint analysis of the data from the five centers showed that the plasma Elafin concentration in Pso was significantly higher than DC (Figure 2F), with an AUC of 0.93 (Figure 2L), and corresponding sensitivity and specificity of 0.86 and 0.94. This indicates that the Elafin kit can not only diagnose Pso but also effectively differentiate between Pso and DC.

### 2.3 Evaluation of Psoriasis Severity

**Figure 3:**
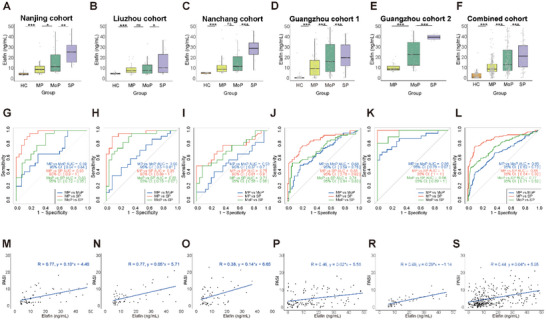
Detection of plasma Elafin detection in Pso of different severity. A–F) Plasma Elafin concentrations significantly differ among MP, MoP, SP, and HC in the five individual cohorts (Nanjing, Liuzhou, Nanchang, Guangzhou Cohort 1, Guangzhou Cohort 2) and the combined cohort. G–L) ROC curve analyses demonstrate that the CLIA kit effectively discriminates Pso with different severities in the five cohorts and the combined analysis. M–S) Significant positive correlations between Elafin concentrations measured by the CLIA kit and PASI scores were observed in all five centers and the combined cohort. NS, not significant (*p* > 0.05); *p < 0.05, **p < 0.01, and ***p < 0.001 compared with Wilcoxon test.

Clinically, disease severity is quantified using PASI. Beyond diagnosis, the Elafin assay enables objective stratification of psoriasis severity. At the Hospital for Skin Diseases, Chinese Academy of Medical Sciences, Guangdong Provincial Hospital of Chinese Medicine and Guangzhou Red Cross Hospital, the Elafin measurement results for severe psoriasis (SP) patients were significantly higher than those for moderate psoriasis (MoP) patients (*p* < 0.01), and the measurement results for MoP were significantly higher than those for mild psoriasis (MP) patients (*p* < 0.001) (**Figure** **3**A,D,E). At Dermatology Hospital of Jiangxi Province and Liuzhou Worker's Hospital, the Elafin measurements for SP were significantly higher than those for MoP, while there was no significant difference between MoP and MP (Figure 3B,C). In all results from the five centers, the Elafin measurements for SP were significantly higher than those for MoP (*p* < 0.001), and the measurements for MoP were also significantly higher than those for MP (*p* < 0.001) (Figure 3F). At the five centers, the AUC for MP vs MoP ranged from 0.53 to 0.86, for MoP vs SP ranged from 0.71 to 0.96, and for MP vs SP ranged from 0.75 to 1.00 (Figure 3G–K). In all data, the corresponding AUC values for MP vs MoP, MoP vs SP, and MP vs SP were 0.68, 0.77, and 0.88, respectively (Figure 3L) (**Table** **7**). To further account for the ordinal nature of severity classification, we performed an ordinal logistic regression analysis, which demonstrated that plasma Elafin levels were significantly and positively associated with psoriasis severity (OR = 1.025, 95% CI: 1.019–1.032, *p* < 0.001). The trend observed was consistent with the ROC pairwise comparisons, supporting the robustness of the findings. At the five centers, the measurements of the kit were significantly positively correlated with the PASI score, with R values of 0.77, 0.77, 0.41, 0.46, and 0.89 (*p* < 0.001) (Figure 3M–R). In all data from the five centers, the measurements of the kit were significantly positively correlated with the PASI score, with an R value of 0.44 (*p* < 0.001) (Figure 3S). Multiple linear regression analysis also showed that the positive correlation between PASI and Elafin remained significant after adjusting for confounding factors of age, treatment status, and gender (*p* <0.001), while the independent effects of age, treatment status, and gender were not significant (Table S3, Supporting Information). Thus, the assay not only stratifies disease severity but also provides a quantitative tool for longitudinal monitoring of psoriasis activity.

**Table 7:** Diagnostic performance of plasma Elafin CLIA for psoriasis stratified by disease severity.

	AUC [95% CI]	Threshold (ng/mL)	SEN [95% CI]	SPC [95% CI]
Pso / HC + DC	0.94 (0.92–0.95)	6.01	0.86 (0.82–0.87)	0.95 (0.92–0.97)
MP / MoP	0.68 (0.62–0.73)	22.12	0.41 (0.34–0.49)	0.89 (0.83–0.93)
MoP / SP	0.77 (0.71–0.82)	49.79	0.56 (0.47–0.63)	0.87 (0.81–0.92)

### 2.4 Full‑Course Management of Psoriasis

**Figure 4:**
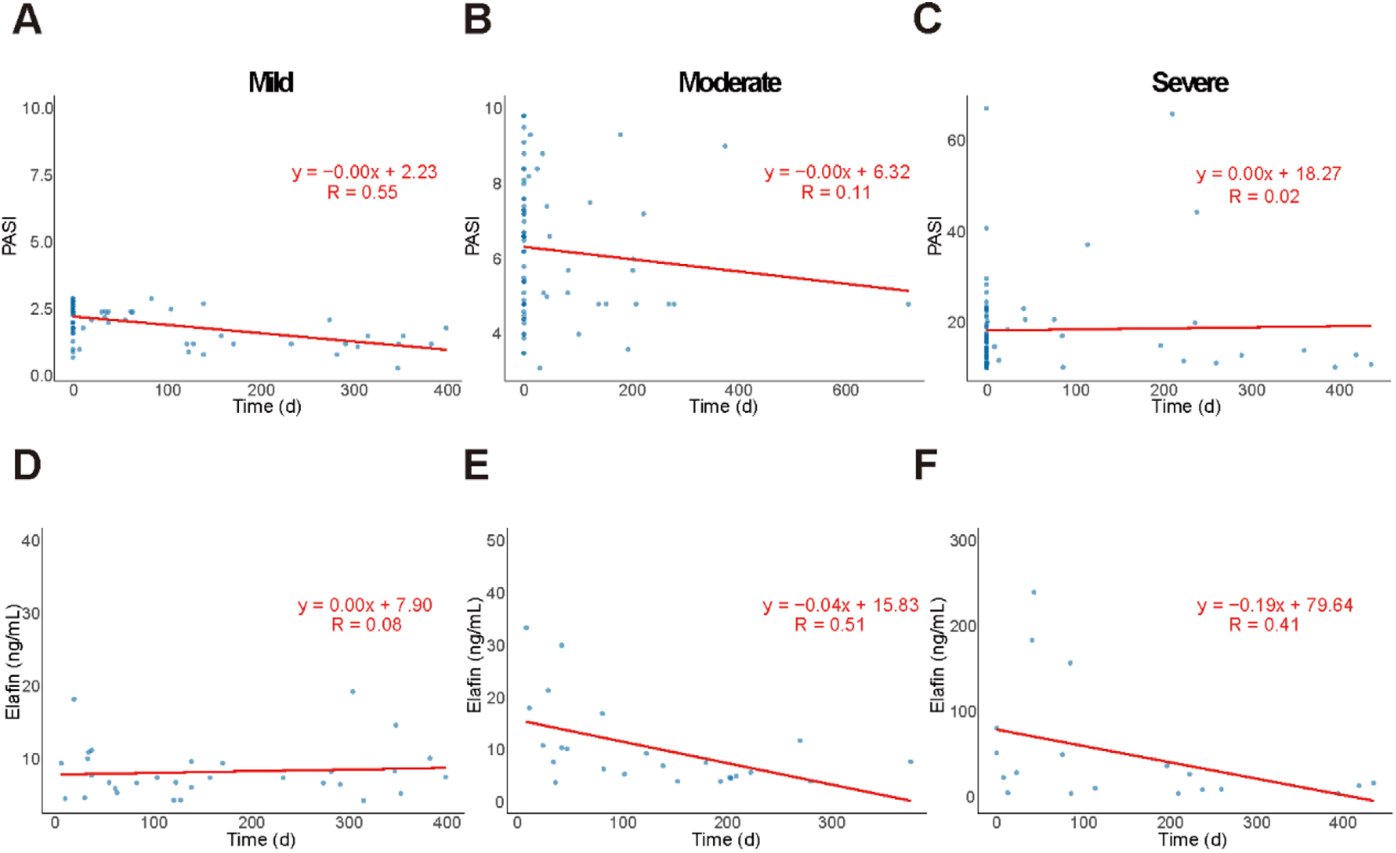
The plasma Elafin CLIA enables full‐course monitoring of psoriasis treatment. A–C) PASI scores in MP, MoP, and SP showed no significant changes with increasing time since the last treatment. D) Plasma Elafin concentrations in MP did not significantly change over time since the last treatment. E,F) Plasma Elafin concentrations in MoP and SP gradually decreased with increasing time since the last treatment.

Currently, there is no rapid and straightforward method to assess treatment efficacy in Pso. Outcomes are evaluated solely through clinical observation of external signs and PASI, which is subjective, complex, and influenced by physician judgment. Moreover, PASI for MP, MoP, and SP showed no significant change over the course of treatment. Although the correlation coefficients were 0.55, 0.11, and 0.02, the slopes of the fitted lines were all 0.00 (**Figure** **4**A–C). In contrast, our kit provides an effective, easy‐to‐use tool for assessing psoriasis severity and enables comprehensive management throughout the treatment course. In MP, Elafin levels remained stable over time (Figure 4D). However, in MoP and SP, Elafin concentrations decreased progressively with treatment duration, with R values of 0.51 and 0.41, respectively (Figure 4E,F). Thus, the kit offers a simple and rapid means for monitoring treatment, as Elafin measurements decline steadily during therapy.

## 3 Discussion

As a prevalent inflammatory skin disease, psoriasis currently lacks rapid, objective, and high‐throughput diagnostic and monitoring tools. Previous studies have proposed Elafin as a biomarker for psoriasis,^[^ ^13^ ^]^ and our prior research confirmed a positive correlation between plasma Elafin levels and PASI scores.^[^ ^10^ ^]^ However, existing ELISA assays remain confined to research settings without multicenter validation, restricting their clinical adoption.

We developed a CLIA reagent kit for plasma Elafin detection using automated analyzers, comprehensively evaluating its analytical precision, accuracy, linear dynamic range, and resistance to common interferents—all meeting rigorous performance standards. Multicenter clinical validation demonstrated the assay's effectiveness in differentiating Pso from both HC and DC, with Elafin levels strongly correlating with PASI scores. Longitudinal analysis further revealed decreasing plasma Elafin with prolonged therapy, underscoring the assay's utility for real‐time monitoring of treatment response.

Compared to traditional ELISA methods, our CLIA reagent kit implemented on automated platforms offers rapid, automated, and high‐throughput detection, facilitating large‐scale clinical screening and more convenient psoriasis diagnosis.^[^ ^31^, ^32^, ^33^ ^]^ Unlike conventional visual assessment methods,^[^ ^5^ ^]^ our CLIA reagent kit effectively mitigates diagnostic confusion with diseases such as pityriasis rosea and atopic dermatitis.^[^ ^7^ ^]^ Moreover, our assay enables objective real‐time assessment of disease severity and therapy monitoring, circumventing invasive histopathological examination.^[^ ^7^, ^28^ ^]^ Although PASI comprehensively evaluates psoriasis severity through erythema, scaling, and skin thickness across various body regions,^[^ ^34^, ^35^ ^]^ it remains subjective, labor‐intensive, and impractical for continuous monitoring.

Among the discordant cases, most patients had received systemic therapy within the past year, mainly including Secukinumab, Methotrexate, Cyclosporine, and steroids. Secukinumab, an IL‐17A inhibitor, blocks IL‐17A binding to its receptor, thus reducing downstream production of pro‐inflammatory cytokines and chemokines.^[^ ^36^, ^37^ ^]^ IL‐17 induces keratinocytes to release cytokines such as TNF‐α, VEGF, and IL‐8; notably, TNF‐α is a primary inducer of endogenous Elafin production.^[^ ^38^ ^]^ Therefore, Secukinumab treatment likely suppresses cytokines such as TNF‐α, reducing plasma Elafin levels.^[^ ^39^ ^]^ Similarly, Methotrexate, a folic acid antagonist, inhibits cytokines including IL‐1, IL‐2, IL‐4, IL‐8, INF‐γ, and TNF.^[^ ^40^, ^41^ ^]^ Cyclosporine modulates T cell subsets, inhibits T cell proliferation, and suppresses the release of mast cell mediators.^[^ ^39^ ^]^ Consequently, these medications may collectively reduce Elafin expression, explaining the lower plasma levels observed in discordant cases (Figure S3A–C, Supporting Information). In particular, accuracy was lower at Liuzhou Worker's Hospital, likely due to differences in therapeutic regimens. At the Hospital for Skin Diseases, Chinese Academy of Medical Sciences, and Dermatology Hospital of Jiangxi Province, most patients were treated with topical ointments and traditional Chinese medicines; Secukinumab was administered only in cases with poor long‐term response. In contrast, Liuzhou Worker's Hospital primarily employed topical ointments combined with systemic therapies such as Methotrexate and Ebastine, both of which have immunosuppressive, anti‐inflammatory, and anti‐proliferative properties (Figure S3D, Supporting Information).

Beyond Elafin's diagnostic utility, elucidating its role in psoriasis pathogenesis reveals potential therapeutic implications. Elafin sits at the intersection of immune regulation and tissue remodeling, acting as both a protective anti‐protease and a marker of persistent inflammatory stress. Its selective upregulation in hyperproliferative keratinocytes, coupled with its strong correlation with local severity and overall PASI scores, underscores its involvement in the core pathological processes of hyperkeratosis and chronic inflammation. The pathological feedback loop involving microbial imbalance, AMP (Elafin) secretion, neutrophil recruitment, and keratinocyte hyperproliferation suggests that therapeutic approaches aimed at enhancing the beneficial anti‐protease activity of Elafin or modulating its upstream regulatory pathways may disrupt the vicious cycle of inflammation and hyperproliferation. Strategies such as modulating microbial community balance, targeting CRABP2‐mediated transcriptional regulation, or using Elafin levels to guide therapy decisions could open new avenues for personalized psoriasis management. These findings provide a robust rationale for future clinical research on Elafin as both a biomarker and a potential therapeutic target.^[^ ^20^ ^]^ Future research investigating Elafin as a therapeutic target may complement current treatment modalities and contribute to personalized medicine approaches in psoriasis management.

This study has several limitations that should be acknowledged. First, the relatively poor AUC value (0.63) for differentiating between mild and moderate psoriasis indicates that the assay has limited ability to distinguish early stages of disease severity. This limitation may partly reflect the small differences in systemic inflammation between these two groups, especially in patients with very limited skin involvement, which reduces the discriminatory power of circulating biomarkers. Furthermore, our severity classification relied solely on PASI, which may be influenced by subjective clinical judgment; incorporating additional metrics such as BSA and DLQI in future studies may improve the sensitivity and specificity of stratification across early disease stages. Second, although Elafin levels showed a significant correlation with PASI scores, the overall correlation was only moderate (r = 0.49). Subgroup analyses revealed substantial inter‐center variation, with correlations ranging from 0.41 to 0.89, suggesting that heterogeneity in patient characteristics, treatment practices, and diagnostic standards across centers may have attenuated the overall association. Previous single‐center studies, including our own,^[^ ^20^ ^]^ have reported stronger correlations, indicating that Elafin retains considerable potential as a monitoring biomarker when confounding factors are minimized. Future research with larger, more homogeneous patient cohorts and standardized clinical assessments will be necessary to enhance the robustness, sensitivity, and generalizability of our findings.

We successfully developed the first CLIA reagent kit for rapid diagnosis and monitoring of psoriasis using automated chemiluminescence analyzers, validated in a multicenter cohort of 614 cases. The kit effectively distinguishes psoriasis from urticaria and atopic dermatitis, while also accurately assessing disease severity. Nonetheless, additional improvements in assay sensitivity could enable more accurate quantification of plasma Elafin. Future studies involving similar conditions, such as pityriasis rosea and eczema, are also warranted to further validate the clinical utility and specificity of the assay.

## 4 Conclusion

We developed and validated a CLIA reagent kit for quantifying plasma Elafin levels using automated chemiluminescence analyzers, enabling high‐throughput psoriasis diagnosis, objective severity assessment, and real‐time therapeutic monitoring. This CLIA reagent kit addresses the critical unmet need for rapid clinical testing in psoriasis, paving the way for precision therapy.

## 5 Experimental Section

### Demographic Characteristics of Participants

This multicenter observational study was conducted at five hospitals: Hospital for Skin Diseases, Chinese Academy of Medical Sciences; Dermatology Hospital of Jiangxi Province; Liuzhou Worker's Hospital; Guangdong Provincial Hospital of Chinese Medicine and Guangzhou Red Cross Hospital. All participants were consecutively enrolled from January 2022 to June 2022 following standardized recruitment protocols. Based on the anticipated assay sensitivity of 0.85, specificity of 0.95, and allowable error of 0.05, a minimum requirement of 196 positive cases and 73 negative cases (total 269) was calculated. Accounting for a 10% dropout rate, the recruitment target was set at 296, leading to enrollment of 731 participants. Participants were consecutively enrolled from dermatology outpatient clinics and routine health examination centers to minimize selection bias. Eligible participants of any age or sex with at least 500 µL of available plasma were enrolled if they met one of the following criteria: clinically confirmed diagnosis of psoriasis according to the “Guideline for the Diagnosis and Treatment of Psoriasis in China (2023 Complete Edition)”, healthy individuals without psoriasis or other dermatological conditions confirmed by clinical examination; or clinically confirmed diagnosis of control disease according to established diagnostic criteria. All participants provided written informed consent. Samples were excluded if they failed to meet collection or storage requirements per protocol, were contaminated during processing, lacked proper ethical approval, had incomplete or missing clinical background data, had insufficient sample volume (<500 µL), or if consent was withdrawn. Of the 755 initially enrolled participants, 20 samples were allocated for assay development, and 735 samples entered the formal study. After excluding 4 samples where the sample size was insufficient due to operational errors, 731 participants were included in the final analysis (Figure S4, Supporting Information).

The cohort comprised healthy controls (HC, N = 112), psoriasis patients (Pso, N = 466) categorized by severity—mild (MP, N = 164), moderate (MoP, N = 158), and severe (SP, N = 144), and disease controls (DC, N = 153) including urticaria (N = 78), eczema (N = 47), atopic dermatitis (N = 2), seborrheic dermatitis (N = 19), pityriasis rosea (N = 6), parapsoriasis (N = 1) (**Table** **8**). According to the “Guideline for the Diagnosis and Treatment of Psoriasis in China (2023 Complete Edition)”, patients with PASI <3 were classified as MP, those with 3 ≤ PASI <10 as MoP, and those with PASI ≥10 as SP.^[^ ^42^ ^]^ The PASI scores of the participants will be evaluated simultaneously with the acquisition of plasma. All participants, including Pso, DC, and HC, provided informed consent after full disclosure of study details. The clinical research protocol underwent ethical review and received approval from the institutional review boards of the participating hospitals (Hospital for Skin Diseases, Chinese Academy of Medical Sciences: 2021–037; Dermatology Hospital of Jiangxi Province: 2021‐016‐01; Liuzhou Worker's Hospital: PJ202144; Guangdong Provincial Hospital of Chinese Medicine: B2012‐53–01; Guangzhou Red Cross Hospital: 2024‐436‐02). The clinical trial was conducted from January 14th to June 28th, 2022.

**Table 8:** Demographic characteristics of clinical cohorts in five independent hospitals.

	HC (N = 112)	DC (N = 153)	Pso (N = 466)		
			MP (N = 164)	MoP (N = 158)	SP (N = 144)
Age	37.64 ± 13.90^a)^	39.73 ± 13.87	42.47 ± 15.14	45.05 ± 16.17	46.24 ± 15.39
Sex (%)					
Male	35 (31.25)	61 (39.87)	111 (67.68)	107 (67.72)	107 (74.31)
Female	77 (68.75)	92 (60.13)	53 (32.32)	51 (32.28)	37 (25.69)
PASI score (%)					
PASI<3	/	/	164 (35.19)	/	/
3≤PASI<10	/	/	/	158 (33.91)	/
PASI≥10	/	/	/	/	144 (30.90)
Cohort (%)					
Hospital for Skin Diseases, Chinese Academy of Medical Sciences	14 (13.46)	16 (15.38)	26 (25.00)	24 (23.08)	24 (23.08)
Dermatology Hospital of Jiangxi Province	14 (14.00)	14 (14.00)	23 (23.00)	25 (25.00)	24 (24.00)
Liuzhou Worker's Hospital	14 (14.00)	14 (14.00)	24 (24.00)	24 (24.00)	24 (24.00)
Guangdong Provincial Hospital of Chinese Medicine	70 (22.58)	58 (18.71)	62 (20.00)	59 (19.03)	61 (19.68)
Guangzhou Red Cross Hospital	0 (0.00)	51 (43.59)	29 (24.79)	26 (22.22)	11 (9.40)

^a)^ Data in the table are presented as mean ± SD.

### Chemiluminescence Immunoassay (CLIA)

First, 50 µL of plasma sample was mixed with 50 µL of anti‐human Elafin antibody‐coated magnetic microspheres (sheep polyclonal antibody) (Heyuan Biotechnology, Guangzhou, China) and incubated at 37 °C for 15 min. During this period, Elafin in the sample bound to the antibody‐coated magnetic microspheres. The mixture was subsequently washed three times with 100 mmol L^−1^ MES buffer (Darui Biotechnology, Guangzhou, China). After washing, 50 µL of acridinium salt‐labeled (NSP‐DMAE‐NHS) (Helisense, Xiamen, China) anti‐human Elafin Antibody (mouse monoclonal antibody) (Heyuan Biotechnology, Guangzhou, China) was added to the samples and incubated at 37 °C for 10 min to form immune complexes. Following a second wash step, pre‐trigger solution (H_2_O_2_, pH <2.0) and trigger solution (NaOH, pH >13.0) were introduced to initiate the chemiluminescence reaction. Finally, to validate the assay's performance across different instruments, the emitted light was measured in relative light units per second (RLU/s) using two automated CLIA analyzers: DR‐CL2000 (Darui Biotechnology, Guangzhou, China) and Shine i1910 (IncreCare, Shenzhen, China).

### Preparation and Validation of Elafin Standards

For the recovery test, the 2.5 mg mL^−1^ standard Elafin solution (Heyuan Biotechnology, Guangzhou, China) was diluted to 150 ng mL^−1^ as Sample A and 5 ng mL^−1^ as Sample B. First, Sample B was tested three times to obtain the average value. Subsequently, 50 µL of Sample A was added to 450 µL of Sample B to prepare the mixed sample (A+B), which was then tested three times to calculate the average value. The recovery rate R was computed using the formula:

(1) $$R=\frac{c\times \left(V_{0}+V\right)-c_{0}\times V_{0}}{V\times c_{s}}\times 100\%$$

*_R_*: Recovery rate

*_V_*: Volume of sample A solution

*V*_0_: Volume of sample B solution

*_c_*: The average concentration of sample B after adding A

*c*_0_: The detected concentration of sample B

*c*_s_: The concentration of sample A

A 2.5 mg mL^−1^ standard Elafin solution was diluted to 25 ng mL^−1^ as quality control sample C1 and 100 ng mL^−1^ as quality control sample C2 for repeatability experiments. Measurements were taken on the same day and after storage at 2–8 °C for 0, 5, 7, 8, and 9 days. The 2.5 mg mL^−1^ standard Elafin solution was further diluted with a diluent to create solutions of 200, 100, 50, 10, 2.5, and 0.8 ng mL^−1^, with each concentration measured in triplicate to generate the standard curve. For the high‐dose hook effect detection, Elafin antigen (Heyuan Biotechnology, Guangzhou, China) was added to the diluent to prepare Elafin solutions at concentrations of 0, 5, 20, 50, 100, 200, 400, 800, and 2000 ng mL^−1^. The limit of blank (LoB) was calculated from 20 replicates of zero‐concentration samples using the formula LoB = Mean + 1.645 × SD, resulting in a LoB of 0.069 ng mL^−1^ for both instruments. The limit of detection (LoD) was calculated from 25 replicates of a 0.8 ng mL^−1^ sample using the formula LoD = LoB + DSβ (where DSβ is the median minus the 5th percentile), yielding 0.096 ng mL^−1^ for the DR‐CL2000 and 0.099 ng mL^−1^ for the Shine i1910; the higher value was taken as the final LoD. The limit of quantification (LoQ) was determined according to CLSI EP17‐A2 guidelines by evaluating the precision and accuracy of 25 replicate measurements at the 0.8 ng mL^−1^ concentration level. The LoQ was established as the lowest concentration where both relative bias and coefficient of variation (CV) were within ±20%, with no more than 3 results exceeding this range. Based on these criteria, the LoQ was confirmed to be 0.8 ng mL^−1^.

### Anti‐Interference Experiment

STB was dissolved in the sample diluent to prepare a 20 mg mL^−1^ solution, which was then added to plasma samples to adjust the concentration to 25 mg dL^−1^. HB was similarly dissolved in sample diluent to form a 500 mg mL^−1^ solution and added to plasma samples to achieve a final concentration of 0.5 g dL^−1^. TG were directly added to plasma samples to set their concentration at 1500 mg dL^−1^. RF raw material was introduced into plasma samples to adjust the RF concentration to 600 IU mL^−1^, while HAMA raw material was added to reach a final concentration of 500 ng mL^−1^. Plasma samples modified with 25 mg dL^−1^ STB, 0.5 g dL^−1^ HB, 1500 mg dL^−1^ TG, 600 IU mL^−1^ RF, and 500 ng mL^−1^ HAMA were subsequently measured.

### Data Statistical Analysis

Statistical analysis and visualization of all data were performed using R (v4.3.1). To verify whether the data followed a normal distribution, the Shapiro–Wilk test was used. For normally distributed data, a *t*‐test was used, while for non‐normally distributed data, the Wilcoxon test was applied to analyze statistical significance between two groups, with *p* < 0.05 considered significant. For correlation analysis, Pearson's or Spearman's method was used for normally or non‐normally distributed data, respectively. Multiple linear regression analysis was performed to assess the relationship between PASI and Elafin while adjusting for potential confounding factors, including age, treatment status, and gender. The standard curve was generated using linear fitting, and the hook effect curve was fitted using a logarithmic model. The pROC package (v1.18.5) was used to plot ROC curves and calculate the area under the ROC curve (AUC) and corresponding 95% confidence intervals (CI). The Youden index was calculated as the sum of the true positive rate (TPR) and specificity (TNR) minus 1, and the threshold corresponding to the maximum Youden index was used to calculate sensitivity, specificity, and the 95% CI. For multicenter data analysis, heterogeneity testing was performed using Cochran's Q test and I^2^ statistics to assess the appropriateness of pooled analysis.

## Conflict of Interest

The authors declare no conflict of interest.

## Supporting information



Supporting Information

## Data Availability

Data sharing is not applicable to this article as no new data were created or analyzed in this study.
