# Supplementary material for: A Multicenter Validation of a Fully Automated Chemiluminescence Immunoassay for Plasma Elafin in Psoriasis Diagnosis and Severity Assessment
Source: Adv Sci (Weinh). 2025 Sep 30;12(47):e11535. doi: 10.1002/advs.202511535 (PMC12713042; doi:10.1002/advs.202511535)
Supplement: Supplementary file 1 — Supporting Information [file ADVS-12-e11535-s001.docx]

Supporting Information

A Multicenter Validation of a Fully Automated Chemiluminescence Immunoassay for Plasma Elafin in Psoriasis Diagnosis and Severity Assessment

*Jingkun Yi, Meng Xu, Ling Han, Zhining Dong, Ruimin Liu, Xiaomei Zhang, Di Hu, Chuanjian Lu* and Xiaobo Yu**


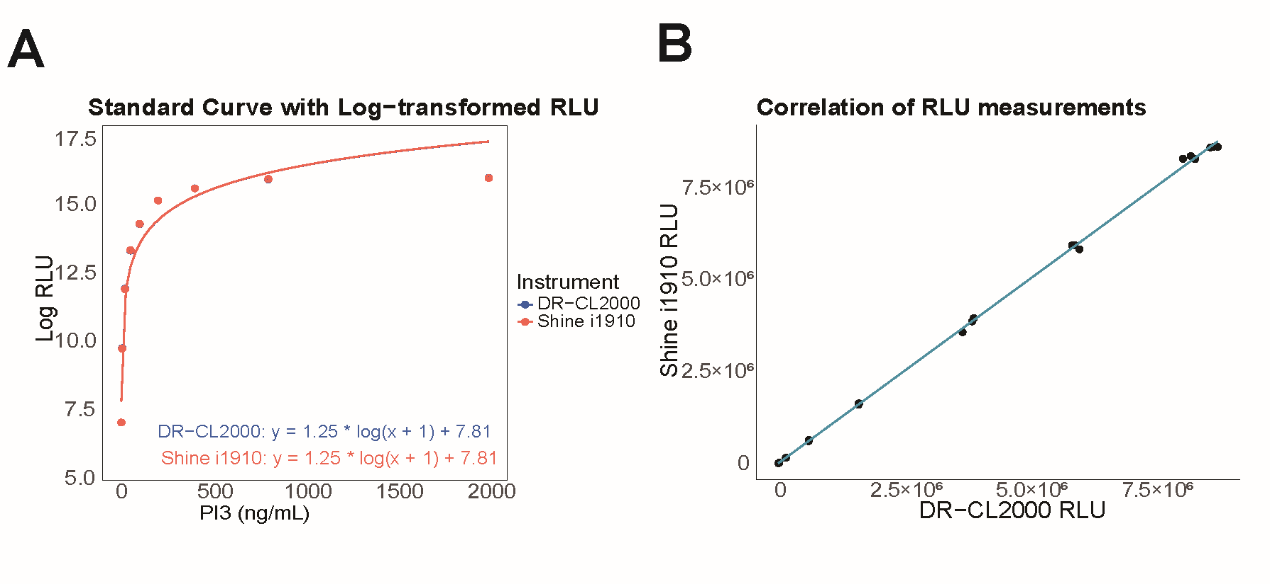


**Supplemental Figure 1.** Absence of the high-dose hook effect in the CLIA kit. (A) CLIA measurements (RLU) did not decrease with increasing plasma Elafin concentrations, indicating no high-dose hook effect in the tested range. (B) Consistent RLU measurements obtained from DR-CL2000 and Shine i1910 analyzers across the high-concentration range.


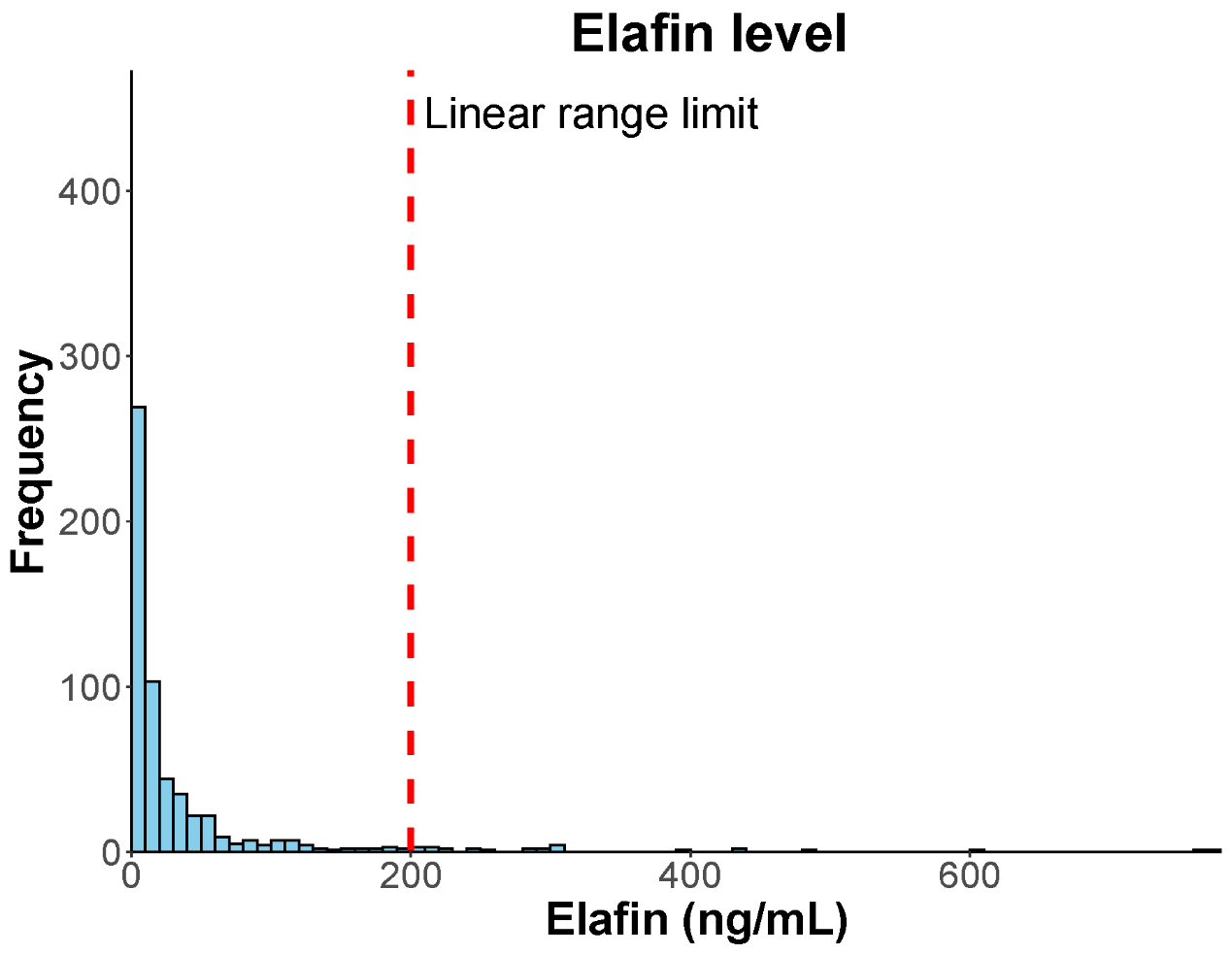


**Supplemental Figure 2.** Plasma Elafin concentrations in patients predominantly within the linear detection range. Most patients had plasma Elafin levels within the linear range of the CLIA kit (<200 ng/mL).

**Supplemental Table 1.** Elafin concentration by age and gender stratification in healthy controls

| Stratification | Subgroup | N | Mean ± SD | Median | Range | P-value* |
| --- | --- | --- | --- | --- | --- | --- |
| Age Groups |  |  |  |  |  | 0.388 |
|  | ≤35 years | 59 | 2.56 ± 2.54 | 1.15 | 0-8.70 |  |
|  | 36-50 years | 32 | 2.70 ± 4.62 | 0.55 | 0-24.60 |  |
|  | >50 years | 21 | 1.61 ± 1.82 | 0.76 | 0-5.35 |  |
| Gender |  |  |  |  |  | 0.770 |
|  | Female | 77 | 2.35 ± 2.28 | 1.40 | 0-7.59 |  |
|  | Male | 35 | 2.59 ± 4.61 | 0.73 | 0-24.60 |  |
| Age × Gender |  |  |  |  |  | 0.407† |
|  | ≤35 years, Female | 36 | 2.68 ± 2.32 | 3.52 | 0-7.59 |  |
|  | ≤35 years, Male | 23 | 2.38 ± 2.90 | 1.07 | 0-8.70 |  |
|  | 36-50 years, Female | 26 | 2.33 ± 2.44 | 0.75 | 0-7.00 |  |
|  | 36-50 years, Male | 6 | 4.29 ± 9.93 | 0.17 | 0-24.60 |  |
|  | >50 years, Female | 15 | 1.57 ± 1.78 | 0.76 | 0-5.35 |  |
|  | >50 years, Male | 6 | 1.71 ± 2.08 | 1.02 | 0-5.35 |  |

*P-values from Kruskal-Wallis test (age groups) and Wilcoxon rank-sum test (gender). †P-value from two-way ANOVA interaction term.

**Supplemental Table 2.** Regression analysis between Elafin and PASI

| Model | Variable | β Coefficient | Standard Error | p-value | R | Significance |
| --- | --- | --- | --- | --- | --- | --- |
| Simple Regression | Elafin | 0.041 | 0.004 | <0.001 | 0.441 | *** |
| Multivariate Regression | Elafin | 0.039 | 0.004 | <0.001 | 0.448 | NS |
|  | Age | 0.030 | 0.022 | 0.169 |  | NS |
|  | Gender | 0.221 | 0.740 | 0.765 |  | NS |
|  | Treatment | 0.943 | 0.734 | 0.200 |  | NS |

Significance codes: *** p<0.001, ** p<0.01, * p<0.05, NS = not significant

**Supplemental Table 3.** Heterogeneity Testing Results

| Metric | Cochran's Q | p-value | I² (%) | Interpretation |
| --- | --- | --- | --- | --- |
| Accuracy | 4.39 | 0.356 | 9 | No heterogeneity |
| Sensitivity | 4.16 | 0.385 | 4 | No heterogeneity |
| Specificity | 0.67 | 0.955 | 0 | No heterogeneity |
| AUC | 1.87 | 0.759 | 0 | No heterogeneity |


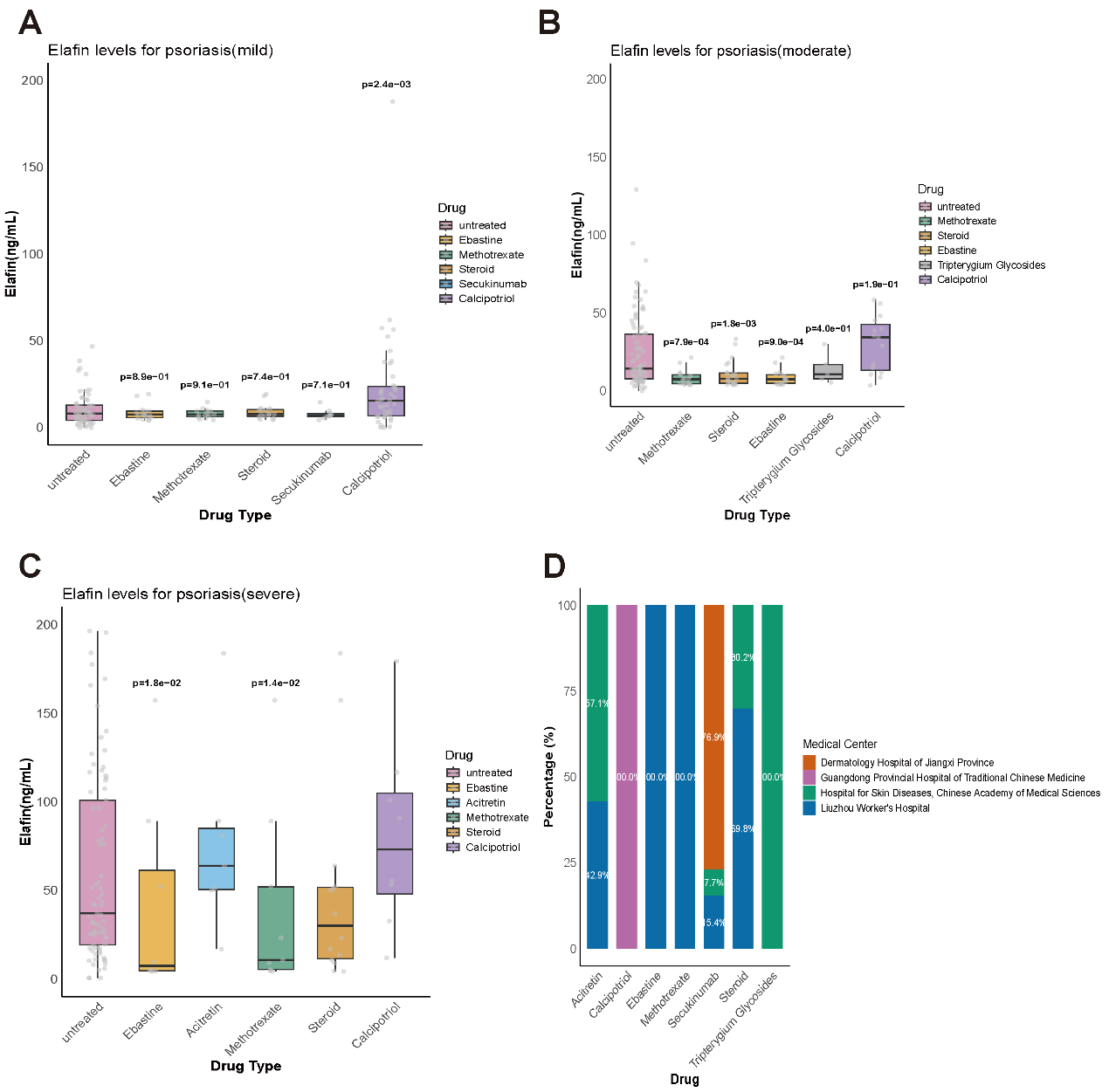


**Supplemental Figure 3.** Medication treatment affects Elafin measurement by the CLIA kit. (A-C) Plasma Elafin concentrations in treated patients were significantly lower than those in untreated patients among mild, moderate, and severe psoriasis groups. (D) Medication preferences for psoriasis treatment differed across the four medical centers.





**Supplemental Figure 4.** STROBE flowchart of study participants.
